# Adipose tissue depot volume relationships with spinal trabecular bone mineral density in African Americans with diabetes

**DOI:** 10.1371/journal.pone.0191674

**Published:** 2018-01-24

**Authors:** Gary C. Chan, Jasmin Divers, Gregory B. Russell, Carl D. Langefeld, Lynne E. Wagenknecht, Jianzhao Xu, S. Carrie Smith, Donald W. Bowden, Thomas C. Register, J. Jeffrey Carr, Leon Lenchik, Barry I. Freedman

**Affiliations:** 1 Department of Internal Medicine, Section on Nephrology, Wake Forest School of Medicine, Winston-Salem, North Carolina, United States of America; 2 Department of Medicine, Division of Nephrology, University of Hong Kong, Hong Kong, China; 3 Division of Public Health Sciences, Department of Biostatistical Sciences, Wake Forest School of Medicine, Winston-Salem, North Carolina, United States of America; 4 Department of Biochemistry, Center for Genomics and Personalized Medicine Research, Center for Diabetes Research, Wake Forest School of Medicine, Winston-Salem, North Carolina, United States of America; 5 Department of Pathology, Wake Forest School of Medicine, Winston-Salem, North Carolina, United States of America; 6 Department of Radiology, Vanderbilt University School of Medicine, Nashville, Tennessee, United States of America; 7 Department of Radiology, Wake Forest School of Medicine, Winston-Salem, North Carolina, United States of America; Nanjing Medical University, CHINA

## Abstract

Changes in select adipose tissue volumes may differentially impact bone mineral density. This study was performed to assess cross-sectional and longitudinal relationships between computed tomography-determined visceral (VAT), subcutaneous (SAT), inter-muscular (IMAT), and pericardial adipose tissue (PAT) volumes with respective changes in thoracic vertebral and lumbar vertebral volumetric trabecular bone mineral density (vBMD) in African Americans with type 2 diabetes. Generalized linear models were fitted to test relationships between baseline and change in adipose volumes with change in vBMD in 300 African American-Diabetes Heart Study participants; adjustment was performed for age, sex, diabetes duration, study interval, smoking, hypertension, BMI, kidney function, and medications. Participants were 50% female with mean ± SD age 55.1±9.0 years, diabetes duration 10.2±7.2 years, and BMI 34.7±7.7 kg/m^2^. Over 5.3 ± 1.4 years, mean vBMD decreased in thoracic/lumbar spine, while mean adipose tissue volumes increased in SAT, IMAT, and PAT, but not VAT depots. In fully-adjusted models, changes in lumbar and thoracic vBMD were positively associated with change in SAT (β[SE] 0.045[0.011], p<0.0001; 0.40[0.013], p = 0.002, respectively). Change in thoracic vBMD was positively associated with change in IMAT (p = 0.029) and VAT (p = 0.016); and change in lumbar vBMD positively associated with baseline IMAT (p<0.0001). In contrast, vBMD was not associated with change in PAT. After adjusting for BMI, baseline and change in volumes of select adipose depots were associated with increases in thoracic and lumbar trabecular vBMD in African Americans. Effects of adiposity on trabecular bone appear to be site-specific and related to factors beyond mechanical load.

## Introduction

Obesity is an important risk factor for the development of type 2 diabetes mellitus (T2D). Higher body mass index (BMI) is also independently associated with cardiovascular disease (CVD) and higher mortality; increases in visceral adipose tissue (VAT) and pericardial adipose tissue (PAT) volumes appear to impart the greatest risk [1,2]. In contrast, obesity may reduce the risk of osteoporosis [3]. However, effects of adiposity on bone mineral density (BMD) remain controversial; some studies failed to support associations between BMD with change in body weight [4,5]. Higher BMD in patients with T2D does not result in lower fracture rates [6]. In a meta-analysis of 16 studies, Janghorbani *et al*. reported a 2.6-fold increased hip fracture risk in men with T2D [7]. In a secondary analysis of 770 women and 1199 men with T2D from three prospective observational studies (the Study of Osteoporotic Fractures, the Osteoporotic Fractures in Men, and the Health, Aging, and Body Composition), Schwartz *et al*. reported that individuals with T2D have higher risk of hip and non-spine fractures compared to age- and BMD-matched controls [8]. Whether these conflicting observations reflect the effect of specific adipose tissue distributions on BMD remains unclear.

Although cross-sectional relationships between volumetric BMD (vBMD) and regional adipose depots have been described [9–13]; few longitudinal studies have been performed. This is particularly true in the understudied African American population. These studies are relevant because African Americans generally have lower volumes of VAT and PAT and higher volume of subcutaneous adipose tissue (SAT) compared to European Americans despite generally higher BMI in African American women compared to European American women and similar BMI in men [14,15]. African Americans also have lower rates of osteopenia and osteoporosis than European Americans, and higher proportions of recent African ancestry (*e*.*g*., lower proportions of European ancestry) are associated with higher BMD [16]. The present study assessed baseline and longitudinal relationships between VAT, SAT, PAT, and inter-muscular adipose tissue (IMAT) volumes with the change in thoracic and lumbar vertebral vBMD in African Americans with T2D. Results may help identify relationships between adipose depot specific change and bone mineral density change which may help to illuminate pathways linking the processes. Study participants were in the African American-Diabetes Heart Study (AA-DHS), an intensively phenotyped cohort for indices influencing bone health [17]. Analyses considered multiple covariates, including BMI, kidney function, and medications.

## Materials and methods

### Study cohort

Between 2007 and 2010, 691 African Americans with T2D were recruited from internal medicine clinics and community advertising in the AA-DHS [17]. The AA-DHS Longitudinal Study subsequently re-examined 300 of these participants after a mean duration of 5.3±1.4 years. These 300 individuals comprise the study group. Identical baseline and follow-up examinations were performed, including interviews for medical history, anthropometric measures, fasting blood testing, spot urine collection, and imaging by quantitative computed tomography (QCT). T2D was defined as a clinical diagnosis of diabetes after the age of 30 years without historical evidence of diabetic ketoacidosis, treatment with oral hypoglycaemic agents or insulin, and/or a fasting blood glucose ≥126 mg/dL, non-fasting blood glucose ≥200 mg/dL, or hemoglobin A1c (HbA1c) >6.5%. Patients with serum creatinine concentration >2 mg/dL were not recruited. The Institutional Review Board at the Wake Forest School of Medicine (WFSM) approved these studies and all participants provided written informed consent.

### Clinical and laboratory measurements

Examinations were conducted in the WFSM Clinical Research Unit. Medications were recorded, including calcium and vitamin D supplements, hormone replacement therapy, bisphosphonates, and oral and inhaled steroids. Height, weight and waist circumference were measured and BMI computed. Laboratory studies included HbA1c, serum creatinine to compute estimated glomerular filtration rate (eGFR), albumin, calcium, phosphorus, 25 hydroxyvitamin D, 1,25 di-hydroxyvitamin D, intact parathyroid hormone (iPTH), and urine albumin:creatinine ratio (UACR) [17].

### Computed tomography measurements

As reported, trabecular vBMD in the thoracic vertebrae (T_8_-T_11_) and lumbar vertebrae (T_12_-L_3_) were determined (mg/cm^3^) using QCT (QCT-5000 software version N-vivo 1.20; Image Analysis Inc., Columbia, KY) [18]. CT examinations were obtained on General Electric systems (Discovery CT750 HD and LightSpeed VCT; GE Healthcare, Waukesha, WI). The vBMD measured by QCT is highly precise with coefficients of variation <1% [19].

SAT, IMAT, VAT, and PAT volumes were measured (cm^3^) from volumetric CT acquisitions to reduce variability related to slice location using Volume Analysis software version 4.2 (Advantage Windows Workstation, GE, Healthcare; Waukesha, WI). A threshold of -190 to -30 Hounsfield units defined adipose containing tissue [20]. VAT, SAT and IMAT measures were centered at L4-L5, covering a volume of 15 mm in the craniocaudal direction. PAT measures were based on the origin of the left main coronary artery, covering a volume of 15 mm cranial and 30 mm caudal. One observer independently analyzed 160 cardiac scan series from 80 participants for volume of PAT. Exams were placed in random order with the observer blinded to other participant information. The two PAT volume measures were highly correlated (Spearman R = 0.99, p<0.0001) and the mean difference in PAT between the first and second scan was 2.11 mL +/- 12.81 mL, not significantly different from zero (p = 0.15). For non-pericardial adipose volumes, one reader provided measurements without assessment of intra- or inter-observer variability.

### Statistical analyses

Sample means and standard deviations were computed for continuous measures and frequencies and proportions were calculated for discrete traits. For variables with highly skewed distributions, median values and interquartile ranges were reported to reflect the central tendency and dispersion. The main predictor variables considered in these analyses were baseline and follow-up volumes of VAT, SAT, IMAT, and PAT for relationships with change in thoracic and lumbar vBMD after an average of 5.3 years of follow-up. Change was defined as the difference between the second visit and baseline vBMD, with this value treated as a continuous outcome. Two linear models were fitted for each combination of changes in the predictor and outcome. The first was a minimally-adjusted model, accounting for baseline vBMD, baseline adipose volume, and the time between measurements. The second was a fully-adjusted model; in addition to the predictors in the minimally adjusted model it included age, sex, smoking, hypertension, BMI, eGFR, use of steroids, hormone replacement therapy (women), calcium and vitamin D supplements. Box Cox transformation was applied on the outcome and residual diagnostic tests were performed to ensure that the linear model assumptions were met.

Association tests were performed at the 0.05 significance threshold for eight tests (4 adipose volumes and 2 vBMD measures), which requires adjustment for multiple testing. However, changes in adipose volumes and vBMD measures are correlated such that adjustment for eight independent tests would be too conservative. The Moskvina and Schimdt approach was applied separately with the 4 change outcomes and 2 predictors to provide two estimates of the effective number of tests (M_1_ for outcomes and M_2_ for predictors) [21]. The M1*M2 product led to the 4.85 value used as the overall effective number of tests. Tests reaching an adjusted p-value <0.01 (<0.05/4.85) were considered statistically significant. Analyses were performed using SAS, version 9.4 (Cary, NC, USA).

## Results

The AA-DHS longitudinal study evaluated 300 unrelated African Americans with T2D who had repeat evaluations after a mean of 5.3±1.4 years; 50% were female. At baseline, the mean±SD age of the cohort was 55.1±9.0 years. Table 1 displays baseline characteristics of the study population stratified by sex. Women had higher BMI and C-reactive protein levels than men, and more women had hypertension and took calcium and vitamin D supplements. One participant took bisphosphonates. S1 Table contains baseline and follow-up demographic and clinical data. S2 Table displays correlations between baseline vBMD and adipose volumes (higher baseline lumbar and thoracic vBMD were positively correlated with SAT and negatively correlated with IMAT).

**Table 1:** Baseline demographic and biochemical parameters of African American-Diabetes Heart Study cohort.

Variable	Baseline Values Stratified by Sex^a^		*P* value
	Female (n = 150)	Male (n = 150)	
Age (years)	55.2 (8.6)	55.0 (9.4)	0.87
T2D Duration (years)	10.2 (6.5)	10.2 (7.9)	0.94
BMI (kg/m^2^)	37.3 (8.0)	32.1 (6.4)	<0.0001
Smoking (% past; % current)	28.7; 20.7	42.0; 24.0	0.01
CKD (%)	38.0	39.3	0.91
CVD (%)	25.3	33.3	0.16
HTN (%)	86.0	75.3	0.028
**Medications**			
Insulin (%)	43.3	40.7	0.73
OHA (%)	77.3	75.3	0.79
Steroids (%)	10.0	4.7	0.12
HRT (%)	28.0	N/A	N/A
Calcium supplements (%)	15.3	3.3	0.0005
Vitamin D supplements (%)	5.3	0.7	0.036
**Biochemistry**			
High-sensitivity CRP (mg/dL)	1.35 (1.93)	0.70 (1.05)	0.0004
HbA1c (%)	8.0 (2.0)	8.2 (2.0)	0.42
25(OH)D (ng/mL)	21.5 (14.1)	18.6 (9.9)	0.041
1,25(OH)_2_D_3_(pg/mL)	49.0 (18.1)	45.4 (17.7)	0.094
iPTH (pg/mL)	61.9 (33.4)	50.3 (25.3)	0.001
Serum calcium (mg/dL)	9.65 (0.42)	9.50 (0.41)	0.0018
Serum phosphorus (mg/dL)	3.67 (0.55)	3.45 (0.59)	0.0009
Urine albumin:creatinine (mg/g)^b^	11.1 (4.0, 48.0)	11.5 (4.3, 56.4)	0.43
eGFR (mL/min/1.73m^2^)	92.6 (22.1)	91.5 (20.2)	0.65

^a^Sample means with standard deviations presented for continuous variables. Discrete traits are described by frequencies and proportions;

^b^Median values with 25^th^ and 75^th^ percentile values presented for urine albumin:creatinine ratio due to skewed distribution;

Abbreviations: BMI, body mass index; CKD, chronic kidney disease; CRP, C-reactive protein; CVD, cardiovascular disease; HTN, hypertension; HRT, hormonal replacement therapy; iPTH, intact parathyroid hormone; OHA, oral hypoglycaemic agent; 1,25 (OH)_2_D_3_ 1,25 dihydroxycholecalciferol; 25(OH)D, 25 hydroxycholecalciferol.

Table 2 displays baseline and follow-up vBMD and adipose volumes, stratified by sex. Mean thoracic and lumbar vBMD decreased over time in both men and women. After mean 5.3 year follow-up, women had significantly higher thoracic vBMD than men. In contrast baseline thoracic and baseline and follow-up lumbar vBMD did not differ significantly between men and women. Adipose tissue volumes increased in all of the regions assessed with the exception of VAT. At baseline and follow-up, volumes of SAT were higher in women, while PAT volumes were higher in men.

**Table 2:** Baseline and follow-up regional bone mineral density and adipose tissue measures.

Baseline and follow-up vBMD and adipose volumes	Values Stratified by Sex		*P* value
	Female (n = 150)	Male (n = 150)	
**Lumbar vBMD**^a^ **(mg/cm**^**3**^**)**			
Baseline	183 (145, 209)	178 (151, 209)	0.83
Follow-up	163 (133, 201)	162 (131, 191)	0.61
Change from baseline	-13 (-24, -3)	-15 (-24, -7)	0.21
**Thoracic vBMD**^a^ **(mg/cm**^**3**^**)**			
Baseline	211 (172, 239)	197 (172, 228)	0.16
Follow-up	193 (158, 223)	176 (148, 203)	0.0065
Change from baseline	-16 (-29, 2)	-23 (-31, -12)	0.0009
**Subcutaneous adipose tissue volume**^b^ **(cm**^**3**^**)**			
Baseline	521 (168)	344 (166)	<0.0001
Follow-up	511 (168)	354 (172)	<0.0001
Change from baseline	-9.1 (98.2)	11.6 (84.3)	0.056
**Inter-muscular adipose tissue volume**^b^ **(cm**^**3**^**)**			
Baseline	12.0 (8.0)	8.9 (6.1)	0.0003
Follow-up	16.3 (9.6)	12.5 (8.9)	0.0004
Change from baseline	4.1 (5.8)	3.8 (6.0)	0.67
**Visceral adipose tissue volume**^b^ **(cm**^**3**^**)**			
Baseline	175.9 (63.9)	187.8 (90.6)	0.20
Follow-up	178.9 (69.0)	187.8 (95.8)	0.35
Change from baseline	3.2 (46.1)	0.4 (62.3)	0.66
**Pericardial adipose tissue volume**^b^ **(cm**^**3**^**)**			
Baseline	83.6 (32.9)	98.2 (50.6)	0.0046
Follow-up	90.9 (37.8)	105.2 (53.8)	0.0082
Change from baseline	6.8 (15.8)	5.1 (20.0)	0.44

^a^Median values with 25^th^ and 75^th^ percentile values;

^b^Sample means with standard deviations;

Abbreviations: vBMD, volumetric bone mineral density.

Table 3 displays longitudinal relationships between mean 5.3 year change in thoracic and lumbar vBMD with changes in each adipose volume. Changes in lumbar and thoracic vBMD were positively associated with the change in SAT (β[standard error] 0.045[0.011], p<0.0001 and 0.040[0.013], p = 0.002, respectively). Change in thoracic vBMD was positively associated with changes in IMAT (0.432[0.198], p = 0.029) and VAT (0.055[0.023], p = 0.016).

**Table 3:** Relationships between five year change in adipose volumes with change in thoracic and lumbar volumetric bone mineral density.

	*Β* Coefficent (Standard Error); *P* value	
Adipose Volume	Thoracic vBMD Change	Lumbar vBMD Change
**Inter-muscular (minimally adjusted)**		
** Change**	0.473 (0.201); **0.019**	0.231 (0.155); 0.14
**Inter-muscular (fully adjusted)**		
** Change**	0.432 (0.198); **0.029**	0.235 (0.160); 0.14
**Subcutaneous (minimally adjusted)**		
** Change**	0.035 (0.013); **0.006**	0.037 (0.010); **0.0003**
**Subcutaneous (fully adjusted)**		
** Change**	0.040 (0.013); **0.002**	0.045 (0.011); <**0.0001**
**Visceral (minimally adjusted)**		
** Change**	0.055 (0.023); **0.016**	0.019 (0.018); 0.28
**Visceral (fully adjusted)**		
** Change**	0.055 (0.023); **0.016**	0.018 (0.019); 0.34
**Pericardial (minimally adjusted)**		
** Change**	0.101 (0.066); 0.13	0.022 (0.052); 0.67
**Pericardial (fully-adjusted)**		
** Change**	0.080 (0.066); 0.22	0.027 (0.057); 0.64

Minimally adjusted model: baseline thoracic/lumbar vBMD and study interval;

Fully adjusted model: baseline thoracic/lumbar vBMD, study interval, age, sex, smoking, hypertension, body mass index, HbA1c, estimated glomerular filtration rate, steroid use, hormonal replacement therapy (women), calcium supplements, and vitamin D supplements (inter-muscular adipose volume and inter-muscular adipose volume change are included only in the lumbar vBMD subcutaneous change model).

Abbreviations: vBMD, volumetric bone mineral density.

Table 4 displays relationships between changes in thoracic and lumbar vBMD with baseline adipose volumes. In models that adjusted for age, sex, smoking, hypertension, BMI, HbA1c, eGFR, and steroid, hormone replacement therapy, calcium and vitamin D supplements, baseline IMAT was significantly associated with the change in lumbar vBMD (0.628[0.157], p<0.0001), with a trend for thoracic vBMD (0.445[0.193], p = 0.021). In contrast, change in PAT (Table 3) and baseline PAT (Table 4) were not associated with changes in either thoracic or lumbar vBMD. Thus, effects of adipose tissue volumes on BMD were region-specific.

**Table 4:** Relationships between baseline adipose volumes with change in thoracic and lumbar volumetric bone mineral density.

	*Β* Coefficent (Standard Error); *P* value	
Adipose Volume	Thoracic vBMD Change	Lumbar vBMD Change
**Inter-muscular (minimally adjusted)**		
** Baseline**	0.630 (0.016); <**0.0001**	0.570 (0.123); <**0.0001**
**Inter-muscular (fully adjusted)**		
** Baseline**	0.445 (0.193); **0.021**	0.628 (0.157); <**0.0001**
**Subcutaneous (minimally adjusted)**		
** Baseline**	0.019 (0.006); **0.003**	0.014 (0.005); **0.003**
**Subcutaneous (fully adjusted)**		
** Baseline**	0.008 (0.013); 0.52	0.017 (0.011); 0.12
**Visceral (minimally adjusted)**		
** Baseline**	0.038 (0.016); **0.015**	0.024 (0.012); 0.053
**Visceral (fully adjusted)**		
** Baseline**	0.031 (0.019); 0.093	0.015 (0.016); 0.35
**Pericardial (minimally adjusted)**		
** Baseline**	0.042 (0.028); 0.14	0.023 (0.022); 0.30
**Pericardial (fully-adjusted)**		
** Baseline**	0.042 (0.034); 0.22	0.007 (0.028); 0.81

Minimally adjusted model: baseline thoracic/lumbar vBMD;

Fully adjusted model: baseline thoracic/lumbar vBMD, age, sex, smoking, hypertension, body mass index, HbA1c, estimated glomerular filtration rate, steroid use, hormonal replacement therapy (women), calcium supplements, and vitamin D supplements (inter-muscular adipose volume is also included only in the lumbar vBMD subcutaneous model).

Abbreviations: vBMD, volumetric bone mineral density.

## Discussion

In fully-adjusted models assessing longitudinal relationships between the changes in volumetric BMD with select adipose depots in African Americans, significant positive relationships were seen between change in SAT with the changes in both thoracic vertebral vBMD and lumbar vertebral vBMD. In addition, strong trends toward positive association were also detected between changes in IMAT and VAT with the change in thoracic vBMD. Higher baseline IMAT was associated with increases in lumbar vBMD developing over an average of 5.3 years, with a trend for increasing thoracic vBMD. These relationships remained after adjustment for age, sex, study interval, baseline BMI, baseline vBMD, HbA1c, kidney function, smoking, hypertension, calcium and vitamin D supplementation, steroid use, and hormone replacement therapy. Although PAT is known to be strongly associated with subclinical coronary artery disease (vascular calcified atherosclerotic plaque), it was not associated with changes in vertebral vBMD [1].

Prior reports evaluating relationships between abdominal adiposity with bone mineralization often employed surrogate indices of VAT, including waist circumference, waist-to-hip ratio, and truncal fat measured using dual-energy x-ray absorptiometry (DXA); and were conducted in diverse and selected populations, including adolescent premenopausal women, postmenopausal women, and men with metabolic syndrome, with conflicting results [4,5,22–24]. More recently, CT and magnetic resonance imaging (MRI) have been used to more directly quantify VAT and SAT. These reports revealed absent (or inverse) cross-sectional relationships between VAT and SAT with BMD [9–13], again in diverse populations, which contrast with the present longitudinal study in African Americans with T2D. It is possible that these discrepancies relate to the cross-sectional nature of prior reports, more accurate assessment of trabecular bone mineral density with QCT in the present report, or sex, age, race, or metabolic differences in study populations. Of the five prior cross-sectional investigations that employed CT or MRI for VAT and SAT, two were in Asian populations, one in a U.S. population (only 2 African Americans were included), and two in which race/ethnicity was not reported [9–13], two involved pre-peak bone mass in adolescents, and two were in obese premenopausal women where endogenous hormonal factors may dominate. Two more recent studies evaluated broader populations of men and women using CT measures of fat depots and bone. Ng *et al*. evaluated 218 women and 291 men over a wide age range (20–97 years) using abdominal CT to assess fat depots and spinal lumbar spine vBMD and found positive associations between SAT and total, trabecular, and cortical vBMD at the spine and other sites, although most associations were not significant after correcting for body weight. VAT was negatively associated with spinal vBMD in young men even after correcting for body weight [25]. More recently, abdominal CT was used to assess adipose tissue and high resolution-pQCT used to assess bone in the distal radius and tibia in 710 non-diabetic subjects (58% women, age 61.3±7.7 years) in the Framingham Osteoporosis Study [26]. VAT was positively associated with trabecular number and vBMD, cortical thickness, and other parameters, mainly in women; however these associations became nonsignificant after adjusting for BMI. SAT and spinal vBMD were not evaluated in this study. Conversely, in a large study of over 8833 clinical CT scans from 7230 patients (46% women) aged 18–65 years, spinal trabecular and cortical x-ray attenuation values (HU) were found to be inversely associated with BMI, SAT, and especially VAT even after adjustment for age and sex, although the associations with fat depots significantly decreased when BMI was a covariate [27]. The basis for the apparent opposite relationships observed using these clinical CT data is unclear.

Positive correlations between adiposity and vertebral BMD may relate to increased load bearing with resultant increased bone mineralization in obese individuals. However, the associations identified in this report were adipose depot-specific and independent from BMI. This suggests that additional mechanisms beyond simple gravitational loading may be involved. Adipose tissue is a source of the aromatase enzyme responsible for the conversion of androgens to estrogens and estradiol, a potent bone protective hormone. Both VAT and SAT have been shown to produce estrone and estradiol in post-menopausal women, with SAT being more efficient in the conversion of estrone to estradiol [28]. Adipokines may also influence bone and other tissues. In 2003, we were the first to demonstrate an inverse relationship between the adipocyte-derived adipokine adiponectin and bone density which was independent of age, sex, and fat mass [18], and later demonstrated inverse relationships with BMD, inflammation, and VAT in the AA-DHS study [18,20]. Adiponectin, which generally decreases in the circulation with increasing adiposity, was inversely associated with VAT volume but not SAT volume in both studies. The potential mechanistic relationship between circulating adiponectin and bone density is not known, although several hypotheses have been put forth, including the idea that falling or low adiponectin could signal the skeleton to increase mass/density to prepare for a future increase in loading [20]. In contrast to adiponectin, leptin increases in circulation with obesity [29], and has central effects on bone metabolism and direct effects on bone cells which may also influence the skeleton. The effects of leptin are complex and include both central and peripheral mechanisms; while hypoleptinemia is associated with lower bone mass, levels above the physiological range do not necessarily increase bone mass [30]. Leptin levels were not measured in this cohort.

Individual adipose depots have other characteristics which may lead to differential effects on cardiometabolic and musculoskeletal metabolism. Increased VAT has been shown to lead to accumulation of macrophages and other immune cells which create a proinflammatory environment and increased adipose depot production of IL-6 and TNF-α which have osteoclastogenic properties potentially increasing bone resorption and turnover. Visceral fat derived IL-6 and TNF-α are also implicated in activation of C-reactive protein production by the liver, another inflammation associated biomarker. Fat depot specific signaling to the skeleton and muscle is an area that needs further investigation.

The AA-DHS cohort represents the largest sample of African Americans with extensive bone and adipose phenotyping; prior genotyping confirmed recent African ancestry. Analyses included longitudinal assessment of the relationships between vBMD with a panel of clinical, imaging, and laboratory measures over mean 5.3 year follow-up. Directly measured volumetric adipose tissue volumes and BMD assessed using CT provide accurate and reproducible measurements [19]. These analyses evaluated the understudied African American population, a group with different distributions of adiposity and bone mineralization compared to European-derived populations [16]. A strength included consideration of a multiple testing penalty, significance was defined by p-values <0.01. Limitations included assessment of only individuals with T2D; hence, results may not extrapolate to individuals without diabetes. In addition, BMD was only measured in the thoracic and lumbar vertebrae, other regions were not assessed, and statistical adjustment was performed for BMI. BMI may not reflect mechanical loading as well as measures such as “percentage body fat” [31]; however, we lack percentage body fat.

In conclusion, this longitudinal investigation in African Americans revealed that changes in thoracic vBMD and lumbar vBMD were positively associated with changes in SAT, with trends seen toward positive association with interval changes in IMAT and VAT, but not pericardial adipose tissue. These results were seen after adjustment for the effect of BMI. The relationships between adiposity and bone are complex and appear to involve factors other than increased mechanical load. Bone-fat relationships may be adipose site-specific and mechanisms underlying these associations require further investigation.

## Supporting information

S1 Table Baseline and follow-up demographic and clinical data. (XLSX)

S2 Table Correlations between baseline vBMD and adipose volumes. (DOCX)
