# Supplementary material for: Adipose tissue depot volume relationships with spinal trabecular bone mineral density in African Americans with diabetes
Source: PLoS One. 2018 Jan 24;13(1):e0191674. doi: 10.1371/journal.pone.0191674 (PMC5783409; doi:10.1371/journal.pone.0191674)
Supplement: S2 Table — (DOCX) [file pone.0191674.s002.docx]

| **Pearson Correlation Coefficients Prob > \|r\| under H0: Rho=0 Number of Observations** | | |
| --- | --- | --- |
|  | **Baseline Lumbar vBMD** | **Baseline Thoracic vBMD** |
| **Baseline pericardial adipose tissue volume (cm^3^)** | -0.072 0.23 | -0.060 0.31 |
| **Baseline visceral adipose tissue volume (cm^3^)** | 0.004 0.95 | 0.062 0.29 |
| **Baseline inter-muscular adipose tissue volume (cm^3^)** | -0.216 0.0003 | -0.170 0.0045 |
| **Baseline subcutaneous adipose tissue volume (cm^3^)** | 0.162 0.0060 | 0.193 0.0010 |

Supplementary Table S2. Relationships between adipose tissue volumes and vertebral bone mineral density at baseline.
